# Efficacy of Short Course of Preksha Dhyana for Functional Abdominal Pain Disorder in a Busy Pediatric Clinic

**DOI:** 10.3389/fped.2021.646686

**Published:** 2021-05-25

**Authors:** Vijay Mehta, Akshay Mehta, Samit Patel, Laura Irastorza, Syed Ahsan Rizvi, Bassam Abomoelak, Naina Mehta, Devendra Mehta

**Affiliations:** ^1^Department of Pediatric Gastroenterology and Nutrition, Orlando Health-Arnold Palmer Hospital for Children, Orlando, FL, United States; ^2^Pediatric Gastroenterology and Nutrition of Tampa Bay, Tampa Bay, FL, United States; ^3^Department of Pediatric Neurology, Orlando Health-Arnold Palmer Hospital for Children, Orlando, FL, United States

**Keywords:** meditation, yoga, functional abdominal pain disorder, irritable bowel syndrome, integrative medicine, Preksha meditation

## Abstract

**Introduction:** Mind body techniques such as meditation improve symptoms in children and adults with IBS. Typical courses, however, are lengthy and difficult to administer. We report our experience with a short course of *Preksha Dhyana* (PD), a child-friendly focused meditation with yoga.

**Method:** Physicians deliver focused meditation while medical assistants taught yoga. Three sessions were administered biweekly with recommendations for daily practice. Pain severity Likert scores were compared with a treatment as usual (TAU) historical control. Anxiety scores were compared from baseline in the PD group.

**Results:** Thirty PD patients aged 9–17 (20 female) and 52 consecutive TAU group aged 5–17 (33 female) were reviewed. The biweekly sessions had high (71%) completion rates. Utilization rates of PD were similar to TAU despite added sessions. The PD group had an average time of follow-up of 8.9 ± 9.4 vs. 6.0 ± 3.9 months in the TAU group (*p* = 0.522). Changes in pain scores from baseline showed improvement in the PD group, 0.67 ± 0.13 vs. TAU 1.39 ± 0.11 (*p* = 0.0003). In the PD group, anxiety scores improved significantly from baseline (0.5 vs. 1, *P* < 0.001). Pain improved in 93% (28/30) and resolved in 47% (14/30).

**Conclusion:** A short course of PD was successfully embedded in a busy pediatric office without additional staffing. The approach proved cost-effective without increasing overall healthcare utilization and showed significant benefits over TAU. Pending RCT confirmation, this offers a cost-effective method to incorporate mind–body techniques into a pediatric office practice.

## Introduction

Functional abdominal pain disorders (FAPDs), including functional abdominal pain (FAP), irritable bowel syndrome (IBS), and functional dyspepsia (FD), are complex disorders characterized by chronic abdominal pain without organic or morphologic etiology. Functional bowel disorders are a common cause of illness-related absenteeism and substantially affect the quality of life (1).

Children and adolescents with FAPDs are more likely to miss school, refrain from normative activities, require care, and exhibit psychological difficulties compared with healthy children (2, 3). It is likely that FAPD takes a particular toll on the academic and social functioning of adolescents and young adults. Fear of pain and/or diarrhea may severely limit adolescent and young adult patient's willingness to attend school and social outings. Such fear further exacerbates symptoms, and over time, FAPD symptoms coupled with the fear of pain can evolve into a vicious cycle in young people (4).

FAPDs often overlap resulting in low sensitivity for questionnaire and classification (5). In ROME IV, there is more emphasis on functional bowel disorders constituting a spectrum of disorders from functional abdominal pain to IBS. IBS itself has three different forms depending on stooling patterns: constipation predominant (IBS-C), diarrhea predominant (IBS-D), or alternating stooling pattern (IBS-M) (6, 7). IBS remains difficult to treat with conventional approaches, including lifestyle changes, diet, cognitive behavioral therapy, and hypnosis, not always available or successful (8). The current understanding of the pathophysiology involves altered brain–gut interaction, along with dysbiosis and altered gut signaling (9). Thus, mind–body approaches, such as yoga and meditation, hold promise in addressing the symptoms and quality-of-life concerns of IBS patients (4).

Yoga consists of *asanas* (body postures) and *pranayama* (prescribed breathing patterns). Yoga combined with meditation has the potential to impact a patient's physical and psychological health. Yoga is commonly practiced to reduce stress and pain (10). Given that patients with IBS are at relative risk for mood disorders, anxiety, and neuroticism, practicing yoga shows promise to ameliorate psychological distress in patients and further downstream effect of pain pathways (11). Randomized studies in patients with IBS and FAP that utilized yoga as treatment have shown promise compared to standard therapy (12–15). Though evidence for the effectiveness of mind–body approaches is promising, methods to integrate them into routine care, and especially outpatient clinic settings, are needed (16). Interestingly, the acceptance and integration of complementary medicine with conventional treatment are growing among patients along with providers (17).

Incorporating yoga and meditation into a regular clinic has been challenging, not least because of the need for trained therapists, costs, and the number of sessions [Bar (18), Ross (19)]. Indeed, reported costs for each 1-h session can be >$100, and insurance coverage remains difficult. Cost—benefit analysis is frequently utilized when deciding on an intervention. However, cost-effectiveness analysis has also been used as a measurement to identify an intervention that may have more benefits at a lower cost or a lower cost with at least as much benefit (20).

The use of yoga and meditation in a clinical setting in pediatrics has been proposed (21), but the incorporation of yoga and meditation in a pediatric subspecialty outpatient clinic has not been reported. In 2016, we developed an integrative medicine clinic (IMC) in a busy pediatric gastroenterology clinic to help manage patients with chronic gastrointestinal disorders including functional abdominal pain syndromes. The clinic teaches a meditative practice called *Prekshya Dhyana* (PD). *Prekshya Dhyana* is the practice of meditation and breathing techniques with yoga used to improve one's perception. It has previously been shown to reduce stress, balance the autonomic nervous system, and increase attention possibly through DNA methylation and thus RNA expression [(22); Abomoelak et al., under review^1^]. We had previously found benefit over a short course of 8 weeks and felt that utilizing a smaller time frame would aid in compliance while not adding to cost burden (23).

Our aim is to assess the feasibility of implementing this novel yoga and meditation intervention within the typical limits of clinic visits in a busy practice and its impact on pain and anxiety in children with IBS.

## Methods

This 2-year study includes a combination of a prospective cohort who underwent the *Preksha Dhyana*–based intervention and historical cohort controls who received treatment as usual (TAU) over the same timeline. The first year of the historical controls are children with FAPD enrolled in a separate observational study where data were collected prospectively while receiving TAU. The second-year control group was obtained by a chart review using our EMR. Inclusion for this study was FAPDs identified by history, physical exam, and laboratory tests with normal endoscopy. The type of FAPD based on ROME criteria was noted, including FAP, FD, IBS-C, IBS-D, and IBS-M. The study groups were managed with an integrative medicine protocol. Thirty patients who met criteria for FAPDs completed a minimum of 4 weeks (two in-person sessions), and 52 patients were in the control group. Both TAU and PD groups received dietary education such as avoiding processed foods with high fructose/carbohydrate, while the use of medications was left to the discretion of the provider. Medications were stratified into daily (cyproheptadine, amitriptyline, SSRI/SNRI) or as needed (Levsin, Bentyl, IBguard).

### Integrative Medicine Clinic Protocol

**Figure 1:**
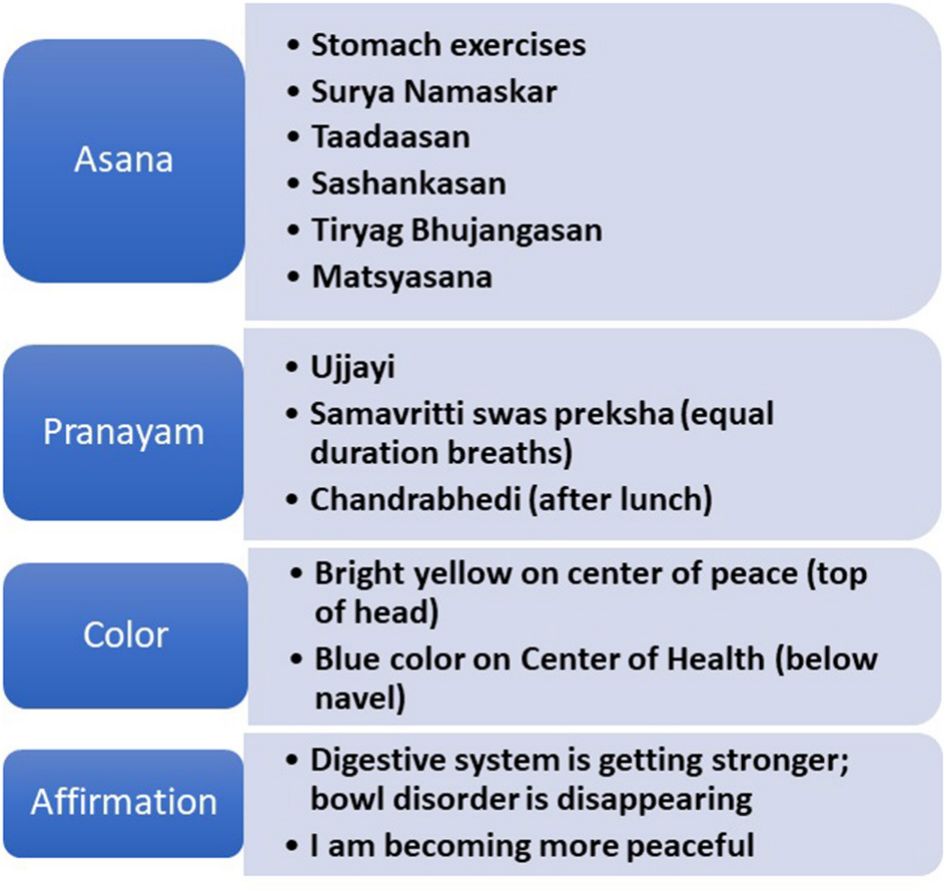
List of poses, breathing, and medication techniques utilized during sessions. Reproduced by kind permission of Meditation 'Science' Conferences 'LLC' Copyrighted '&' proprietary 'material.

The integrative medicine sessions included *yoga and meditation* in a calm environment with soft music and aroma candles. Yoga and *pranayama* (breathing exercises) in the form of *Prekshya Dhyana* were conducted by trained medical assistants or coordinators, including RNs and physicians. Sessions lasted 60 min with a greater emphasis on poses (Figure 1) that involve the abdomen including those ideal to expel gas. To aid with anxiety, a common comorbidity, breathing techniques including diaphragm breathing (*kapalabati)* and alternate nostril breathing (*anulom vilom)* were emphasized in addition to the poses listed. The physician conducted the focused meditation portion using guided imagery that also served to focus on the abdomen and areas of chronic discomfort. We utilized patient–physician interaction for guided meditation to aid in the patient/parent comfort. Overall, the physician spent about 15 min, excluding charting. Patients were expected to continue *Prekshya Dhyana* at home daily at a fixed time and were recommended to use the breathing techniques specifically for a few minutes as needed during episodes of pain or stress. Adherence to these techniques was assessed on follow-up office visits. Overall goals were to help with increased flexibility, relaxation, and resilience and provide individuals with a coping mechanism during times of stress allowing them to continue tasks or activities, especially school related.

### Cost-Effective Strategy

Our program was developed over several Plan, Do, Study, Act (PDSA) cycles. By training several regular clinic staff, medical assistants, and nurse coordinators, sustained scheduling was feasible (cost $1,200 per staff). As a side benefit, staff skill sets were broadened with improved satisfaction. Prior to COVID-19, we combined two or three patients sensibly matched for age per session. Clinics were conducted late afternoons in an underutilized office space. Completion was deemed as practice for 4 weeks (two in-office sessions) out of 6 weeks if the child, parent, and therapist agreed. During their last session, the routine to be used regularly was reduced to about six items, totaling 12 min. This time was close to the amount adolescents felt feasible. The poses (two or three), breathing (two or three), and focused meditation (minimum of one) components were selected by the child in a partnership model with the physician, and the typical time in the day to carry out the session for each child was also defined. Additional breathing exercises when feeling pain or stress were selected. This was presented as the patient's own personal, written “prescription,” signed by both the child and the physician, with at least 1 day a week dedicated to the full 45-min program being the expectation.

We used guidelines from the American Academy of Family Physicians (24). With our local payors, we confirmed that the use of individual E and M code would be accepted including combined time when seen as a group with the physician. We tried various options available, including using group codes, as well as codes for education and nutrition counseling.

### IM Clinic Details and Evaluation

A Questionnaire on Pediatric Gastrointestinal symptoms, Rome IV version (QPGS-RIV), was administered prior to each IMC session. This is a validated questionnaire and commonly used for FGID, including validation of criteria (25, 26).

To assess clinical efficacy, we focused on changes in pain severity, stress/anxiety, and clinic utilization as recorded in the EMR from baseline to last visit. Specifically, changes in pain scores from baseline to last available visit were derived from the EMR or study records for the prospective control cohort and standardized on a Likert scale of resolved, improved, unchanged, worsened, or required urgent visits.

Anxiety was assessed by a chart review of self-reported history similarly using a Likert scale (none, mild, moderate needing counseling, severe needing an additional medication, and extreme requiring withdrawal from school or hospitalization), and changes from baseline were evaluated.

### Statistical Analysis

Age is expressed as mean with standard deviation, M (SD). Differences in age and gender were conducted with Mann-Whitney *U*-test. FAPD proportions were tested with the Pearson's *X*^2^ (with Yate's continuity correction). The proportion of pain medication utilization was compared with Fisher's exact test. Time periods for clinical follow-up and pain scores were compared with Mann–Whitney *U*-test. All *p*-values were derived from two-sided tests, and the results were determined statistically significant when *p* < 0.05. Cost and billing information was descriptive and estimated given the variability of the payor mix.

## Results

Thirty *Prekshya Dhyana (*PD) patients aged 9–17 (20 female) and 52 treatment-as-usual (TAU) group aged 5–17 (33 female) were reviewed (Table 1). The specific FAPD diagnosis is included in the table, which varied between five different types. There was no difference in the subtype frequency of FAPD, *X*^2^ (4, *N* = 82) = 0.9688, *p* > 0.05. The biweekly sessions had high (77%) completion rates (mean 4.3 weeks or 2.15 visits) with continued in-home daily sessions using guided handouts.

**Table 1:** Demographics including age, gender, and primary FAPD diagnosis.

	**PD (*n* = 30)**	**TAU (*n* = 52)**	***P*-value**
Age (years)	14 (2.3)	13 (3.5)	0.447
Female	20	33	0.815
IBS-C	7	13	0.914
IBS-D	7	12	
IBS-M	2	5	
FD	7	8	
FAP	7	14	

The time between the first and the third visit in the prospective control group (*n* = 30) was not significantly different to the time from endoscopy to the last recorded visit in the retrospective group (*n* = 22) (*p* > 0.05) (Table 2). Indeed, comparing either group with the PD group for all variables showed a similar pattern as when combined (data not shown). Medication use was available for 15 of 30 prospective controls and combined with the retrospective cohort. Of the TAU group, 30% (11) utilized daily medication vs. 46.7 % (14) in PD (*p* > 0.05). As-needed medication was used in 46% (17) of the TAU group compared to 60% (18) in the PD group (*p* > 0.05).

**Table 2:** Comparison of follow-up times and pain improvement between retrospective and prospective controls.

	**Prospective control**	**Retrospective control**	***P*-value**
Time period for clinic follow-up (months)	5.6 (5.8)	6.4 (1.3)	0.06
Follow-up Course pain improvement	1.6 (0.5)	1.7 (1.3)	0.83

**Figure 2:**
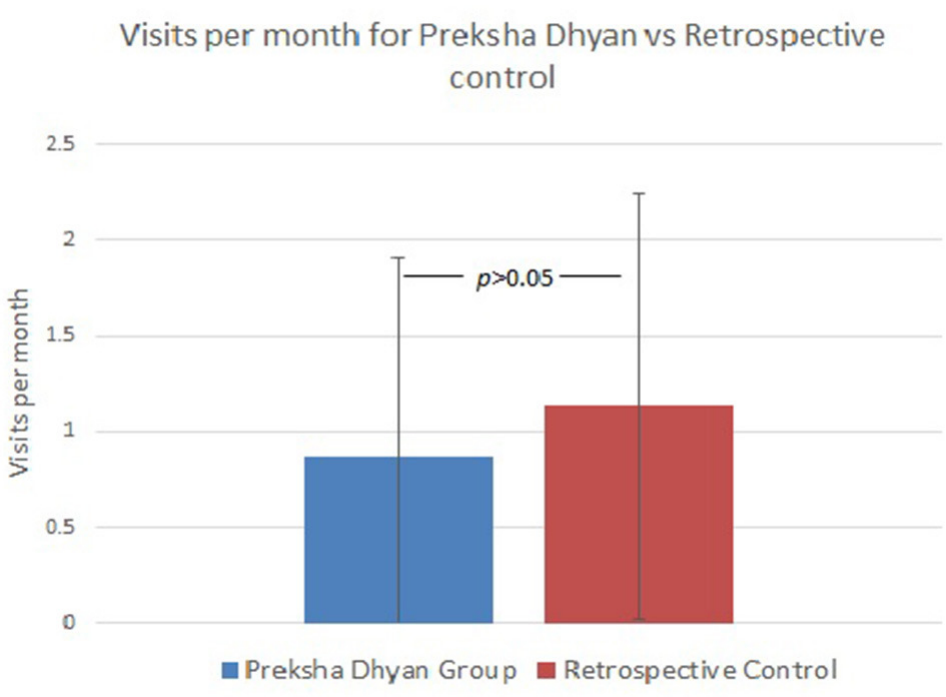
Comparison of visits per month between Prekshya Dhyan and Retrospective Control.

The two control groups were combined (TAU) and compared to the PD group. The PD group had an average time of follow-up of 8.9 ± 9.4 vs. 6.0 ± 3.9 months in the TAU group (*p* = 0.522). Changes in pain scores from baseline showed improvement in the PD group 0.67 ± 0.13 vs. TAU 1.39 ± 0.11 (*p* = 0.0003) (Table 3). Clinic visits per month was 0.9 ± 1.0 in the PD group vs. 1.1 ± 1.1 in the retrospective group (*p* > 0.05) (Figure 2).

**Table 3:** Demonstrates the difference in follow-up times and improvement in pain scores between the PD and TAU.

	**PD**	**TAU**	***P*-value**
Time period for clinic follow-up (months)	8.9 (9.4)	6 (3.9)	*P* = 0.522
Pain score improvement	0.67 (0.13)	1.39 (0.11)	*P* = 0.0003

More patients in the PD group (46%) had resolution of pain vs. the TAU group (12%) (*p* < 0.001) (Table 4). Pain improved in 93% (28/30) of the participants.

**Table 4:** Shows the resolution of pain between the PD and TAU groups.

	**PD**	**TAU**	**Total**	***P*-value**
Pain	16	46	62	
No pain	14	6	20	
Total	30	52	82	<0.001

In terms of anxiety, the IM group had higher baseline anxiety scores (*N* = 29, mean 1.5, SD 1,1) compared with controls (*N* = 28, mean 0.71, SD 0.9, Mann–Whitney *U, p* < 0.01). This suggests a referral bias where those with anxiety were more likely to be identified for referral to the integrative clinic as 76% (22/29) compared to 43% (12/28) in the retrospective control group, though not reaching significance (*P* > 0.05).

Despite a tendency for higher baseline anxiety scores, the PD group showed significant improvement (1.48 vs. 0.86, *P* < 0.001) while the control group improvement was marginal (from 0.71 vs. 0.68, n.s.).

Additionally, among the patients in the PD group, there was improvement in symptoms such as constipation from 18 patients to 10, diarrhea from 11 to 2, and nausea from 21 to 7 patients. Adherence to techniques on follow-up was missing in part as many did not need further follow-up.

### Costs/Revenue

Family costs ranged from 0 copayments to $50 copayments per session. Some patients with private insurance had an additional $16-$25 for deductibles. A maximum for a 6-week course was estimated to be $200 for private insurance, though the majority, based on ~50% Medicaid and managed care payor mix, paid none or < $100 total out of pocket expenses.

Training office staff cost were $4,800. Physician use of CPT 99212-99214 as appropriate was covered by all payors, even in a group setting. As the techniques were applied to other conditions also such as IBD, CF, and GERD, the expenses incurred in training were recouped within 12 months. Registered dietitians used CPT code 97804, which was often not paid as was considered bundled. Education and training for patient self-management involving a standardized curriculum (98960–98962) for our trained “therapists” were not agreed to by our payors and was also considered bundled. Finally, an option we did not get clarity from our payors was as follows: code 99078, which describes physician educational services in a group, and although this is covered in some states, we ultimately did not attempt using them.

## Discussion

We successfully implemented *Prekshya Dhyana* (PD) as part of an integrative medicine program in a busy, private pediatric gastroenterology practice. We had previously shown PD benefits in school children with ADHD and learning-disabled students (27–29). We more recently found significant improvement in short-term memory, cognitive function, and positivity with reduced negativity in college students (23). However, this was over 8 weeks and would likely pose significant hurdles in clinical practice. We used PDSA cycles to settle on a model of three visits over 6 weeks, with ongoing adoption daily at home. This addressed several concerns regarding cost, time off work, and school and also allowed those partly familiar with yoga to finish in two sessions within 4 weeks.

We compared changes from baseline values and noted significant improvements in measures for both pain and anxiety. We found that pain scores improved both based on our Likert scale, but also in pain resolution as reported by participants in the PD group. Similarly, participants in the PD group also reported improved anxiety scores. When compared with the TAU group, this improvement is especially significant for pain measures, as baseline anxiety scores were already lower in the TAU group, perhaps suggesting a referral bias for more anxious patients to the integrative medicine clinic. In line with this, we did note more patients in the PD groups utilized daily and as-needed medication, though not statistically significant. These patients may have warranted medication due to combined severity of anxiety and pain.

The majority of the patients in the PD group had sessions over 4 weeks, with at least two in-office visits and showed benefit. Utilizing the minimum number of sessions to produce effect will likely reduce costs in the long run and improve compliance. Based on our experience, physicians were reimbursed for E and M codes, including their involvement in group sessions. Extra training of core clinical staff was recouped. Cost to the families was substantially lower than reported with comparable programs [Ross (19)]. In our study, we found that the time and visits per month were no different between the PD and control groups, and thus no difference in the overall cost of care.

A study conducted by Nyrop et al. (30) regarding the costs of health care for functional gastrointestinal disorders found annual costs of $5,455 for IBS, $6,434 for functional diarrhea, $7,912 for chronic constipation and $7,950 for abdominal pain including out of pocket expenses. Pediatric costs often include costs for families including loss of employment. In a pediatric group diagnosed with functional gastrointestinal disorders, the average annual costs were $6104.30 (31). A similar study evaluating costs for adolescents with chronic pain found a median of $6,770 (32).

The use of complementary medicine has been rising due to low cost as well as lack of effectiveness of conventional medicine. Interestingly, other practices that have incorporated alternative medicine found that patients were practicing on their own prior to initiation of clinics and found success among patients and healthcare providers (19). In previous studies, 40–50% of patients utilized complementary medicine for functional disorders (33, 34). Studies in adults and children have shown that mind–body techniques (MBTs) have efficacy in IBS, along with pain modification likely related to the brain–gut axis (9, 35). Our retrospective cohort study confirms that a readily applicable technique can be applied in most outpatient settings with a brief intervention of 4-6 weeks.

### Limitations

This study does have limitations in being retrospective and lacking standardized questionnaires for anxiety and pain. However, the intent of the study was to see if our short intervention was cost-effective compared to those treated as usual in a busy clinical practice. Routine questionnaires for stress, anxiety, and depression were not part of the standard of care and so were not justified in this setting. Long-term use of techniques taught was not systematically followed as intended as many reported resolution of symptoms at the end of the intervention and did not need to return for follow-up. A follow-up telephone survey was not part of the IRB approved study but would have been helpful. Dietary counseling was provided to all patients with functional abdominal pain disorder, regardless of group, based on principles of following recommended dietary allowances. To what extent this was followed or played in symptom improvement between groups was not specifically evaluated. Medication use was slightly higher in the PD group, but not significantly so. This does support the use of the mind–body approach as complementary to TAU. However, we cannot exclude medication resulting in the improvement of pain and anxiety as opposed to PD.

A prospective randomized study with age and gender matched of patients that are enrolled in IMC compared to TAU would provide more accurate information with regards to utility. In addition, the use of a validated questionnaire for diagnosis along with follow-up of clinical metrics such as pain and anxiety would give more complete comparison between groups, while careful logging of adherence to the recommendations would be important to measure.

True cost was not obtained as it is difficult to estimate for patients, though overall visit numbers suggest that this was not increased in those participating in integrative medicine clinic.

Despite the limitations, we were able to demonstrate a positive impact of the program and how a simple structure can be incorporated into a busy private practice.

## Conclusion

We showed utility and feasibility in incorporating *Preksha Dhyana* into a busy private clinic as complementary care for patients that met the criteria for the spectrum of FAPD including IBS, with significant improvements shown in the patient's pain and anxiety. While further, more controlled studies are necessary to confirm the effects of a short course of meditation and yoga, this study provides a promising and feasible direction for future practice.

## Data Availability Statement

The original contributions presented in the study are included in the article/Supplementary Material, further inquiries can be directed to the corresponding author/s.

## Ethics Statement

The studies involving human participants were reviewed and approved by Orlando Health-Arnold Palmer Medical Center Institutional Review Board. Written informed consent from the participants' legal guardian/next of kin was not required to participate in this study in accordance with the national legislation and the institutional requirements.

## Author Contributions

DM devised the project, the main conceptual ideas, and proof outline. VM and AM wrote the manuscript in consultation with SP, NM, and DM. VM, AM, SP, LI, SR, BA, and DM worked on data entry. VM and DM performed calculations. All authors contributed to the article and approved the submitted version.

## Conflict of Interest

The authors declare that the research was conducted in the absence of any commercial or financial relationships that could be construed as a potential conflict of interest.

## Footnotes

^1^Abomoelak B, Pragya SU, Griswold A, Mehta N, Uddin P, Veeramachaneni P, et al. *Preksha Dhyāna* meditation induces alterations at the transcriptome and DNA Methylation Levels in Novice and Healthy College Students. (2021).

## References

[1] Cain KC Headstrom P Jarrett ME Motzer SA Park H Burr RL. Abdominal pain impacts quality of life in women with irritable bowel syndrome. Am J Gastroenterol. (2006) 101:124–32. 10.1111/j.1572-0241.2006.00404.x 16405544

[2] Varni JW Lane MM Burwinkle TM Fontaine EN Youssef NN Schwimmer JB. Health-related quality of life in pediatric patients with irritable bowel syndrome: a comparative analysis. J Dev Behav Pediatr. (2006) 27:451–8. 10.1097/00004703-200612000-00001 17164617

[3] Hyams JS Burke G Davis PM Rzepski B Andrulonis PA. Abdominal pain and irritable bowel syndrome in adolescents: a community-based study. J Pediatr. (1996) 129:220–6. 10.1016/S0022-3476(96)70246-9 8765619

[4] Naliboff BD Fresé MP Rapgay L. Mind/body psychological treatments for irritable bowel syndrome. Evid Based Complement Alternat Med. (2008) 5:41–50. 10.1093/ecam/nem046 18317547 PMC2249749

[5] Palsson OS Whitehead WE van Tilburg MA Chang L Chey W Crowell MD. Rome IV diagnostic questionnaires and tables for investigators and clinicians. Gastroenterology. (2016). 10.1053/j.gastro.2016.02.014. [Epub ahead of print]. 27144634

[6] Mearin F Lacy BE Chang L Chey WD Lembo AJ Simren M. Bowel disorders. Gastroenterology. (2016). 10.1053/j.gastro.2016.02.031. [Epub ahead of print]. 27144627

[7] Simren M Palsson OS Whitehead WE. Update on Rome IV criteria for colorectal disorders: implications for clinical practice. Curr Gastroenterol Rep. (2017) 19:15. 10.1007/s11894-017-0554-0 28374308 PMC5378729

[8] Sandhu BK Paul SP. Irritable bowel syndrome in children: pathogenesis, diagnosis and evidence-based treatment. World J Gastroenterol. (2014) 20:6013–23. 10.3748/wjg.v20.i20.6013 24876724 PMC4033441

[9] Drossman DA Hasler WL. Rome IV-functional GI disorders: disorders of gut-brain interaction. Gastroenterology. (2016) 150:1257–61. 10.1053/j.gastro.2016.03.035 27147121

[10] Barnes PM Bloom B Nahin RL. Complementary and alternative medicine use among adults and children: United States, 2007. Natl Health Stat Report. (2008) 1–23. 10.1037/e623942009-001 19361005

[11] Ford AC Talley NJ Schoenfeld PS Quigley EM Moayyedi P. Efficacy of antidepressants and psychological therapies in irritable bowel syndrome: systematic review and meta-analysis. Gut. (2009) 58:367–78. 10.1136/gut.2008.163162 19001059

[12] Kuttner L Chambers CT Hardial J Israel DM Jacobson K Evans K. A randomized trial of yoga for adolescents with irritable bowel syndrome. Pain Res Manag. (2006) 11:217–23. 10.1155/2006/731628 17149454 PMC2673138

[13] Taneja I Deepak KK Poojary G Acharya IN Pandey RM Sharma MP. Yogic versus conventional treatment in diarrhea-predominant irritable bowel syndrome: a randomized control study. Appl Psychophysiol Biofeedback. (2004) 29:19–33. 10.1023/B:APBI.0000017861.60439.95 15077462

[14] Brands MM Purperhart H Deckers-Kocken JM. A pilot study of yoga treatment in children with functional abdominal pain and irritable bowel syndrome. Complement Ther Med. (2011) 19:109–14. 10.1016/j.ctim.2011.05.004 21641514

[15] Korterink JJ Ockeloen LE Hilbink M Benninga MA Deckers-Kocken JM. Yoga therapy for abdominal pain-related functional gastrointestinal disorders in children: a randomized controlled trial. J Pediatr Gastroenterol Nutr. (2016) 63:481–7. 10.1097/MPG.0000000000001230 27050045

[16] Thakur ER Shapiro J Chan J Lumley MA Cully JA Bradford A. A systematic review of the effectiveness of psychological treatments for IBS in gastroenterology settings: promising but in need of further study. Dig Dis Sci. (2018) 63:2189–201. 10.1007/s10620-018-5095-3 29744772

[17] Berna F Göritz AS Mengin A Evrard R Kopferschmitt J Moritz S. Alternative or complementary attitudes toward alternative and complementary medicines. BMC Complement Altern Med. (2019) 19:83. 10.1186/s12906-019-2490-z 30961586 PMC6454683

[18] Bar J. Bringing yoga therapy into mainstream health care: lessons from the Cleveland clinic and their relationship to emotional well-being. Int J Yoga Therap. (2013) 67. 24165525

[19] Ross A Williams L Pappas-Sandonas M Touchton-Leonard K Fogel D. Incorporating yoga therapy into primary care: the Casey Health Institute. Int J Yoga Therap. (2015) 25:43–9. 10.17761/1531-2054-25.1.43 26667288

[20] Bergmo TS. How to measure costs and benefits of eHealth interventions: an overview of methods and frameworks. J Med Internet Res. (2015) 17:e254. 10.2196/jmir.4521 26552360 PMC4642791

[21] Simkin D. Meditation and Mindfulness in Clinical Practice. Child and Adolescent Psychiatric Clinics of North America (2014). 10.1016/j.chc.2014.03.002 24975623

[22] Jain V Jain K Shweta S Prajna SC. Yoga-Preksha-Dhyan practice as a cost-effective preventive strategy against aggressiveness in primary school children. Int J Yoga Allied Sci. (2017) 6:106–13.

[23] Pragya SU Mehta ND Abomoelak B Uddin P Veeramachaneni P Mehta N. Effects of combining meditation techniques on short-term memory, attention, and affect in healthy college students. Front Psychol. (2021) 12:607573. 10.3389/fpsyg.2021.607573 33746830 PMC7973112

[24] AAFP. Coding for Group Visits: American Academy of Family Physicians. n.d.Available online at: https://www.aafp.org/family-physician/practice-and-career/getting-paid/coding/group-visits.html (accessed March 29, 2021).

[25] Caplan A Walker L Rasquin A. Development and preliminary validation of the questionnaire on pediatric gastrointestinal symptoms to assess functional gastrointestinal disorders in children and adolescents. J Pediatr Gastroenterol Nutr. (2005) 41:296–304. 10.1097/01.mpg.0000172748.64103.33 16131984

[26] Caplan A Walker L Rasquin A. Validation of the pediatric Rome II criteria for functional gastrointestinal disorders using the questionnaire on pediatric gastrointestinal symptoms. J Pediatr Gastroenterol Nutr. (2005) 41:305–16. 10.1097/01.mpg.0000172749.71726.13 16131985

[27] Pragya U Cordoba G Chui J Mehta N Johnson P Mehta D. Can the cognitive parameters of college students with learning disabilities benefit from using Mahapraan, a breathing based Preksha meditation? J Coll Teach Learn. (2014) 11:169. 10.19030/tlc.v11i4.8854

[28] Mehta S Mehta V Shah D Motiwala A Vardhan J Mehta N. Multimodal behavior program for ADHD incorporating yoga and implemented by high school volunteers: a pilot study. ISRN Pediatr. (2011) 2011:780745. 10.5402/2011/780745 22389788 PMC3263567

[29] Shah D Shah K Mehta S Mehta N Mehta V Mehta V. Peer-mediated multimodal intervention program for the treatment of children with ADHD in India: one-year followup. ISRN Pediatr. (2012) 2012:419168. 10.5402/2012/419168 23316384 PMC3539379

[30] Nyrop KA Palsson OS Levy RL Von Korff M Feld AD Turner MJ. Costs of health care for irritable bowel syndrome, chronic constipation, functional diarrhoea and functional abdominal pain. Aliment Pharmacol Ther. (2007) 26:237–48. 10.1111/j.1365-2036.2007.03370.x 17593069

[31] Dhroove G Chogle A Saps M. A million-dollar work-up for abdominal pain: is it worth it? J Pediatr Gastroenterol Nutr. (2010) 51:579–83. 10.1097/MPG.0b013e3181de0639 20706149

[32] Groenewald CB Essner BS Wright D Fesinmeyer MD Palermo TM. The economic costs of chronic pain among a cohort of treatment-seeking adolescents in the United States. J Pain. (2014) 15:925–33. 10.1016/j.jpain.2014.06.002 24953887 PMC4150826

[33] Kong SC Hurlstone DP Pocock CY Walkington LA Farquharson NR Bramble MG. The incidence of self-prescribed oral complementary and alternative medicine use by patients with gastrointestinal diseases. J Clin Gastroenterol. (2005) 39:138–41. 10.1097/01.mcg.0000177234.36640.68 15681910

[34] Vlieger AM Blink M Tromp E Benninga MA. Use of complementary and alternative medicine by pediatric patients with functional and organic gastrointestinal diseases: results from a multicenter survey. Pediatrics. (2008) 122:e446. 10.1542/peds.2008-0266 18662934

[35] Shah K Ramos-Garcia M Bhavsar J Lehrer P. Mind-body treatments of irritable bowel syndrome symptoms: an updated meta-analysis. Behav Res Ther. (2020) 128:103462. 10.1016/j.brat.2019.103462 32229334

